# Memoirs of the Royal Medical Society at Paris, Vol. III

**Published:** 1784

**Authors:** 


II. *Memoirs of the Royal Medical Society at Paris, vol.* III. *(continued from page* 128.)

MEDICAL *Chemistry.*— 1. *Remarks on the magnesia of Epsom salt. By* M. Macquer. —Although the process for making magnesia is not unknown, yet it has hitherto, we are told, been but little practised in France, so that almost all the magnesia used there has been imported from England. The present paper is intended by its author, to make its uses and the manner of preparing it, more known to his countrymen. In the course of the paper, he takes occasion to correct a mistake in the new edition of his chemical dictionary, where he directs magnesia to be only *slightly* washed.

When

When he publiſhed that work, he was not aware, it ſeems, of the inſolubility of magneſia in boiling water; a fact, for the knowledge of which, we are indebted to Mr. Butini of Geneva *(ſee our 2d vol. p. 358)*; and moreover, he imagined, that the ſalts remaining in the magneſia, after its being ſlightly waſhed, might contribute to its purgative quality; but he is now convinced, that its perfect inſipidity is one of the qualities which ought to recommend it; and, therefore, that it muſt be thoroughly waſhed with boiling water.—2. *Analyſis of columbo root, by* M. Joſſe, *apothecary at Paris.*—3. *Analyſis of the root of John de Lopez, by* the ſame.—4. *Obſervations on a mixture of Peruvian bark, with emetic tartar. By M.* Cornette.—Our author having prepared this medicine, in the proportion of 24 grains of the emetic to an ounce of bark, found it to be a medicine of great efficacy in intermittents. He is of opinion, that the reguline part of the antimony is precipitated by the aſtringent property of the bark.

*Botany.*—*Obſervations on two ſpecies of quinquina, lately diſcovered near Santa Fé in South America. By Meſſieurs* Daubenton, Macquer, Bucquet, de Juſſieu *and* Cornette.—Theſe gen-

 tlemen tlemen were requested by the society to examine two species of bark sent to them from Madrid. The reader will see the result of their observations mentioned in our fourth volume, page 308.

*Medical philosophy.—Inquiries and experiments, relative to the organ of hearing and the propagation of sounds, by* M. Perolle.—This paper has lately been published in a separate form, under the title of *Dissertation Anatomico-acoustique.* (See vol. 4, p. 218.)

*Supplement to the historical part of vol.* II. *for* 1777 and 1778.—1. *On the Cesarean operation.*—M. Chabrol, surgeon at Mezieres, has communicated some observations on the operation he performed there, and of which an account was communicated to the society by M. Hennequin *(see vol.* 4, *p.* 239.) Previous to the operation the parts of generation, we are told, were much irritated and swelled. The first incision, though of considerable length, was not sufficient. Two lateral incisions were made into the uterus. The omentum which protruded at the wound, was reduced, and three flat ligatures were passed through the integuments, to bring the lips of the wound together. During the cure, portions of intestine and omentum omentum appeared at the upper part of the wound, but were reduced.—2. *Supplement to M.* Van Woenfel's *obfervations on the virtues of mufcus pyxioides in the chin cough (fee vol.* IV. *p.* 362).—We have here an account of the manner of adminiftering this remedy. Three drachms of it are directed to be boiled in a pint of water, till it is reduced to ten ounces. This quantity divided into four, fix, or more dofes, and fweetened with fugar, is to be taken every day.

Having finifhed our account of the firft or hiftorical part of the volume, we come now to the fecond part, or MEMOIRS. Under this head we meet with the following papers.

1. *Conftitution of the year* 1779, *at Paris. By* M. Geoffroy.—2. *Sequel of the conftitution of the year* 1779, *with obfervations on the epidemic cough at the end of the year* 1779 *and beginning of* 1780. *By* the fame.—3. *On the epidemic difeafe of Rouvray St. Denis. By* Abbè Teffier.—4. *Hiftorical account of the epidemic dyfentery, which prevailed during the autumn of* 1779 *in moft of the provinces of France. By* M. Caille.—This epidemic, of which we have here a good account, and which, in confequence of neglect and mifmanagement feems to have been extremely fatal in the be-

[illegible], was analogous to the dysentery that prevailed at Nimeguen in 1736 and has been so well described by Degner.—5. *Account of an epidemic disease, which prevailed during the winter of the year 1779, at Bois le Roy near Anet in Normandy, communicated by* M. Galeron, *physician at Jury la Bataille.*—6. *On the diseases which prevailed at Dinan in Britanny, among the English prisoners. By Messrs.* Jeanroy *and* Lalouette.—The disorder here described was a jail fever, which proved extremely fatal. The number of sick, at one time, amounted to eight hundred, and one in fifteen of those who were attacked with it died.—7. *Medical topography of Montmorenci and its environs, by Father* Cotte.—We have here a very accurate account of the situation of Montmorenci;—its latitude and longitude;—its elevation above the Seine at Paris, and above the ocean;—its mineralogical history;—its well water, spring water, and sulphureous mineral water;—diseases that have prevailed there during the last eleven years;—state of its population, from which we learn, that the proportion of deaths annually, is one in thirty-four; that the total number of inhabitants is 1580, and of families 270; that on an average, five children are produced by each marriage; that that the proportion of widows to widowers, is as seven to four; that of unmarried women to unmarried men, as six to four; and that of boys to girls, under fourteen years of age, as ten to nine: that the births of boys and girls, are in the proportion of thirteen of the former to twelve of the latter; that in the burials, the proportion of men to women, is as nine to ten; and that of boys to girls, as fifteen to twelve: so that more females than males die among adults, and more males than females among children.

[ *To be continued.* ]

