# Errata

**Published:** 1784

**Authors:** 


					In. the prefent volume, page 249, line fy, tor medicine read mixture ?,

				

